# A 2‐Min Cytomegalovirus (CMV) Awareness Video Improves Pregnant Women's Knowledge and Planned Adherence to Hygiene Precautions

**DOI:** 10.1111/ajo.70016

**Published:** 2025-03-25

**Authors:** Tanya Tripathi, Jotara Watson, Hayley Smithers‐Sheedy, Kath Swinburn, Natalia Rode, Emma Waight, Annabel Webb, Natasha E. Holmes, Hanako Stump, Antonia Shand, Lisa Hui

**Affiliations:** ^1^ Department of Obstetrics, Gynaecology and Newborn Health University of Melbourne Melbourne Victoria Australia; ^2^ Cerebral Palsy Alliance Research Institute The University of Sydney Sydney New South Wales Australia; ^3^ Department of Infectious Diseases and Immunology Austin Health Melbourne Victoria Australia; ^4^ CMV Australia Sydney New South Wales Australia; ^5^ Faculty of Medicine and Health University of Sydney Sydney New South Wales Australia

**Keywords:** cytomegalovirus, developmental disability, education, pregnancy, primary prevention

## Abstract

**Introduction:**

Congenital cytomegalovirus (cCMV) is a leading infectious cause of life‐long neurodevelopmental disabilities, but public awareness of CMV is low. This study evaluated a short educational video on cCMV for its acceptability and impact on pregnant women's knowledge and planned hygiene adherence.

**Materials and Methods:**

Participants were pregnant women recruited from an Australian tertiary maternity hospital clinic and social media (May 2023 to May 2024). Participants completed online surveys: before the video (T1), immediately after (T2), and 8 weeks later (T3). Linear mixed effects models assessed changes in knowledge and intended adherence to CMV precautions, adjusting for previous CMV education, and parity.

**Results:**

A total of *n* = 296 eligible pregnant women were recruited, *n* = 270 completed the T1 survey and watched the video. Participants (*n* = 270) had a median age of 33 years (range: 18–43 years), 21% were multiparous and 30% had received previous CMV education. Of the 270 participants who completed the T1 survey and viewed the video, 202 (75%) and 109 (40%) completed surveys at T2 and T3 respectively. Adjusted total mean CMV knowledge scores increased significantly between T1 and T2 (+2.38; *p* < 0.001) and remained higher at T3 (+2.14; *p* < 0.001). Self‐reported adherence to hygiene precautions improved from T1 to T2 (*p* < 0.001) and were maintained for four out of five key behaviours at T3. Participants (99%) found the content valuable, and 91% agreed that CMV precautions were “easy” to follow.

**Conclusion:**

A CMV education video is a simple, effective method to improve pregnant women's knowledge and planned adherence to hygiene precautions.

## 1 Introduction

Cytomegalovirus (CMV) is a common herpesvirus that is transmitted through bodily fluids like urine and saliva [1]. In healthy individuals it usually causes mild or no symptoms. It is, however, the leading cause of congenital infections, affecting 0.48% of live births in high‐income and 1.42% in low‐income countries [2], and is a preventable cause of severe neurological disabilities, such as hearing loss, cerebral palsy, and vision impairment [2, 3, 4]. People who have close contact with the children under 3 years of age are considered at an increased risk of infection due to the high rates of CMV excretion in young children [5, 6].

Hygiene practices during pregnancy are effective in reducing CMV transmission [7, 8], and pregnant women value CMV prevention education [9, 10]. Qualitative research involving women with lived experience of congenital CMV during pregnancy highlights strong support for raising public awareness: “I feel women should be told about CMV” and “If you don't tell people, there is no chance in doing the right thing” [11]. National and international authorities including, the Australian Department of Health, and the Royal Australian and New Zealand College of Obstetricians and Gynaecologists (RANZCOG) recommend risk reduction hygiene measures including avoiding contact with children's saliva and urine by handwashing to reduce congenital CMV infection risk [12, 13, 14, 15]. Despite this, public awareness remains low globally [16]. In Australia, only 1 in 6 pregnant women know about CMV, and less than 10% of healthcare professionals report that they routinely provide antenatal counselling about CMV [9].

A 2024 systematic review emphasised the importance of prenatal education and empowering pregnant women to adopt hygiene measures to reduce CMV transmission [17]. Educational tools like brochures, one‐on‐one counselling, and videos can improve CMV awareness [18, 19, 20]. Short engaging videos, may offer a cost‐effective, scalable option though their impact on CMV knowledge and prevention in Australia remains untested. This study aimed to (i) investigate whether viewing a 2‐min educational video on congenital CMV could increase pregnant women's knowledge of CMV and motivation to adhere to recommended hygiene precautions during pregnancy, and (ii) evaluate the acceptability and clarity of the video content.

## 2 Materials and Methods

### 2.1 Study Setting

Eligible participants were individuals who were: currently pregnant, Australian residents with intention to birth in Australia, aged 18 years or older, and able to provide informed consent in English. Participants were recruited through social media channels of a national congenital CMV consumer advocacy group (CMV Association), the individual and institutional social media accounts of the investigator team, and in‐person invitation at the antenatal clinic waiting area of a tertiary maternity hospital in Melbourne, Australia.

The social media campaign utilised the maternity hospital's Facebook and Instagram channels to target Australian women aged 18–40 years, using Meta's pre‐defined categories for interests in motherhood, fatherhood, pregnancy, and parenting. Hospital‐based recruitment involved posters through QR codes linking to the study webpage, and in‐person outreach by study staff in the antenatal clinic waiting room. Participants accessed the Participant Information and Consent Form via REDCap [21], an electronic data capture tool hosted by the University of Melbourne.

### 2.2 Data Collection

After consenting online, participants completed demographic and obstetric history surveys through a REDCap questionnaire, including questions on CMV awareness and information received during their pregnancy.

#### 2.2.1 Pre‐Video Baseline CMV Knowledge and Behaviour Survey (T1)

Following the demographic survey, participants completed an online questionnaire to report their baseline CMV knowledge in a multiple‐choice format. Questions covered CMV transmission, pregnancy risks, and prevention, with knowledge scores ranging from 0 to 6, awarding 1 point per correct response (Appendix Survey S1). Participants with regular contact (≥ 1 day/week) with children under 5 years completed an additional section on CMV risk behaviours, rated on a 5‐point Likert scale from ‘Never’ to ‘Always’. CMV risk behaviour scores ranged from 0 to 25, with higher scores indicating greater adherence to preventive measures (Appendix Survey S1).

#### 2.2.2 CMV Educational Video (Intervention)

After completing the baseline survey, participants were directed to a publicly available congenital CMV video on YouTube. Developed in 2018 by the Cerebral Palsy Alliance in collaboration with clinicians and individuals with lived experience from the CMV Association, the 2‐min 30‐s video provides an overview of CMV, its potential long‐term effects, transmission routes, and recommended hygiene precautions [22]. The video is narrated in English.

#### 2.2.3 Post Video Survey (T2)

Immediately after viewing the CMV educational video, participants completed a follow‐up survey with the same CMV knowledge questions as in T1. The survey also included additional questions about participants' perceptions on the importance of the information provided in the video, the ease of adopting the suggested hygiene behaviours, and their attitudes towards practicing CMV prevention measures (Appendix Survey S2).

#### 2.2.4 8‐Week Post‐Video Survey (T3)

Eight weeks after T2, participants were emailed a link to a third survey, which included the same questions as T1 and T2, along with questions on how often they engaged in behaviours that could increase their risk of CMV infection during pregnancy. The survey aimed to investigate any changes in behaviour in response to the CMV educational video. (Appendix Survey S3).

Adapted from a previous Australian survey assessing pregnant women's knowledge and attitudes about CMV before and after receiving information [23], the surveys were modified by the research team, which included a general practitioner (NR), a maternal fetal medicine specialist (LH), and researchers experienced in patient‐reported outcomes (HSS, LH and KS). A consumer representative (HSt) with lived experience of congenital CMV in pregnancy piloted and provided feedback.

### 2.3 Data Analysis

Descriptive statistics examined participant characteristics and survey responses at each time point. Mean changes in test scores between T1 and T2, and T1 and T3, were estimated using a linear mixed effects model, with test scores as the response, time point as the predictor, and a random intercept for repeated measures. A similar model estimated changes in adherence to CMV hygiene precautions. Prior CMV awareness significantly impacted baseline knowledge scores, while previous births influenced baseline CMV prevention behaviour scores. To account for this these factors were controlled in the model. Changes in participants' agreement with the ease of following CMV hygiene behaviours, stratified by prior birth status, were evaluated using *z*‐test. A *p*‐value of < 0.05 was considered significant. Statistical analysis was performed in *R*, using the Ime4 and ordinal packages. Free text responses at T2 and T3 were analysed using inductive content analysis (ICA) with iterative coding led by TT, with co‐coding by LH [24].

### 2.4 Ethics Approval

The study was approved by the Mercy Health Human Research Ethics Committee on November 22, 2022 (HREC no. 2022‐047).

## 3 Results

### 3.1 Participants

**FIGURE 1:**
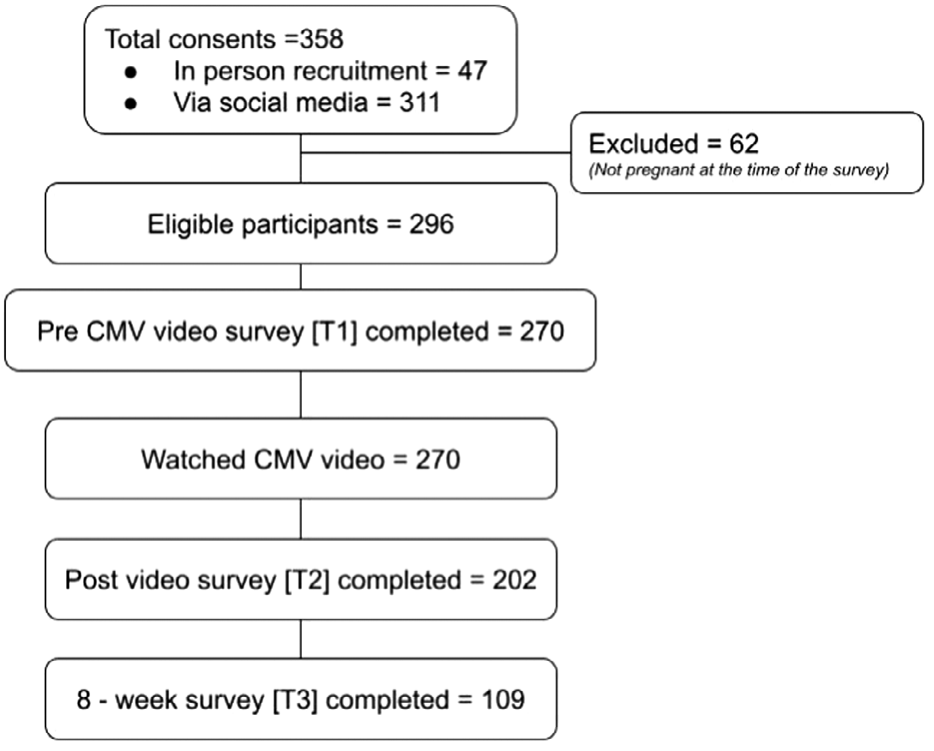
Flow chart of survey participation.

From May 2023 to May 2024, 358 participants consented to the study. Of these *n* = 296 were pregnant and eligible to participate. A total of *n* = 270 participants completed the T1 survey and watched the CMV video and were included in the analysis. Table 1 summarises the demographic and obstetric histories of the participants. Of the 270 eligible women who entered the study, 75% (202/270) completed the post‐video survey at T2, and 40% (109/270) completed the 8‐week follow‐up survey at T3 (Figure 1).

**TABLE 1:** Demographics and obstetrics history (*n* = 270).

*Demographics*	
Age, median (range)	33 (18–43) years
Gestational age, median (range)	20 (2–39) weeks
Birth country	
Australia	216 (80%)
Other	54 (20%)
Preferred language spoken at home	
English	260 (96%)
*Obstetric history*	
Gravidity	
First pregnancy	102 (38%)
Second pregnancy	100 (37%)
Three or more	68 (25%)
Parity (*n* = 168)	
No previous births	23 (14%)
One child	110 (65%)
Two or more	35 (21%)
Model of care	
Shared antenatal care with GP	46 (17%)
Routine hospital based antenatal care	31 (11%)
High risk hospital based antenatal	24 (9%)
Midwifery‐led care	71 (26%)
Private obstetric care	70 (26%)
Private midwife	9 (3%)
Unsure/Undecided	19 (8%)
*Prior CMV knowledge*	
Previously heard of CMV	177 (66%)
Received information/education about CMV	53 (30%)
○ Source of information/education received about CMV	
GP	23 (30%)
Midwife	16 (21%)
Hospital doctor	7 (9%)
Website	7 (9%)
Social media	3 (4%)
Friends and family	5 (7%)
Other	15 [CMV organisation = 1, Brochure provided by midwife = 1, Hospital pamphlet = 1, Fertility specialist = 1, and Book = 1] (20%)

Abbreviations: CMV, cytomegalovirus; GP, general practitioner.

### 3.2 CMV Knowledge

#### 3.2.1 Baseline CMV Knowledge—T1

Two‐thirds of the participants (66%, 177/270) had heard of CMV; however, only 30% (53/177) received information about CMV during their current pregnancy, most commonly from their general practitioner (GP) (30%), or midwife (21%) (Table 1). Participants with prior awareness of CMV demonstrated significantly higher baseline CMV knowledge compared to those without. No significant differences were found for factors, such as Socio‐Economic Indexes for Areas (SEIFA) quintile, maternal age, previous pregnancies or births, or regular contact with children (Table S1).

#### 3.2.2 Change in CMV Knowledge Scores—T1 vs. T2, T1 vs. T3

Participants' adjusted total mean CMV knowledge scores improved significantly between T1 and T2 [+2.38 (95% confidence interval [CI] 2.09–2.67); *p* < 0.001], and between T1 and T3 [+2.14 (95% CI 1.79–2.49); *p* < 0.001]. Analysis of mean score changes for individual CMV knowledge questions showed significant improvements across all six questions (Q1–Q6 Appendix Survey S1) between T1 and T2, as well as between T1 and T3 (*p* < 0.001 for all comparisons).

### 3.3 CMV Prevention Behaviour

#### 3.3.1 Baseline CMV Prevention Behaviour—T1

At T1, 59% (159/270) of participants reported frequent contact with young children and rated the frequency of five activities that increased their CMV infection risk. Of these participants, 87% (139/159) had been pregnant before and engaged in significantly higher frequencies of CMV risk behaviours compared to first‐time pregnant participants. No significant differences were found for other factors, such as SEIFA quintile, maternal age, or prior CMV awareness (Table S2).

#### 3.3.2 Change in CMV Prevention Behaviour—T1 Vs. T2, and T1 vs. T3

At T2, 70% (112/159) of participants who watched the CMV education video and had frequent contact with young children reported their intention to practice CMV prevention measures. After adjusting for birth history, scores showed increased intent to practice CMV prevention at T2 compared to T1 [+4.0 (95% CI 3.5–4.5); *p* < 0.001].

**FIGURE 2:**
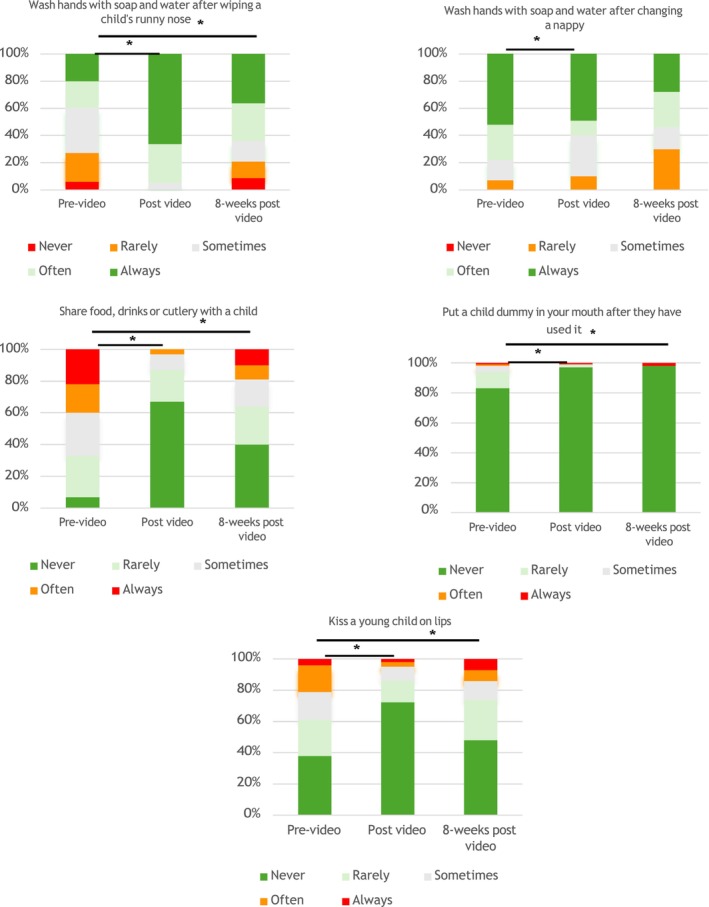
Change in participants' intent to practice CMV prevention measures after watching the CMV education video, and implementation of these behaviours 8 weeks post video. *Represents statistically significant result (*p* < 0.001).

At T3, 54% (109/202) of participants who completed the T2 survey reported their CMV prevention behaviours over the past 8 weeks. Adjusted scores showed sustained improvement in CMV preventative behaviours from T1 to T3 [+1.6 (95% CI 1.0, 2.2); *p* < 0.001]. Adherence to four of five key prevention behaviours—handwashing after wiping a child's nose, avoiding sharing food/drinks, or cutlery, not putting a child's dummy in the mouth, and avoiding kissing a child on the lips—was maintained at T3 (Figure 2).

### 3.4 Acceptability of CMV Prevention Recommendations

**FIGURE 3:**
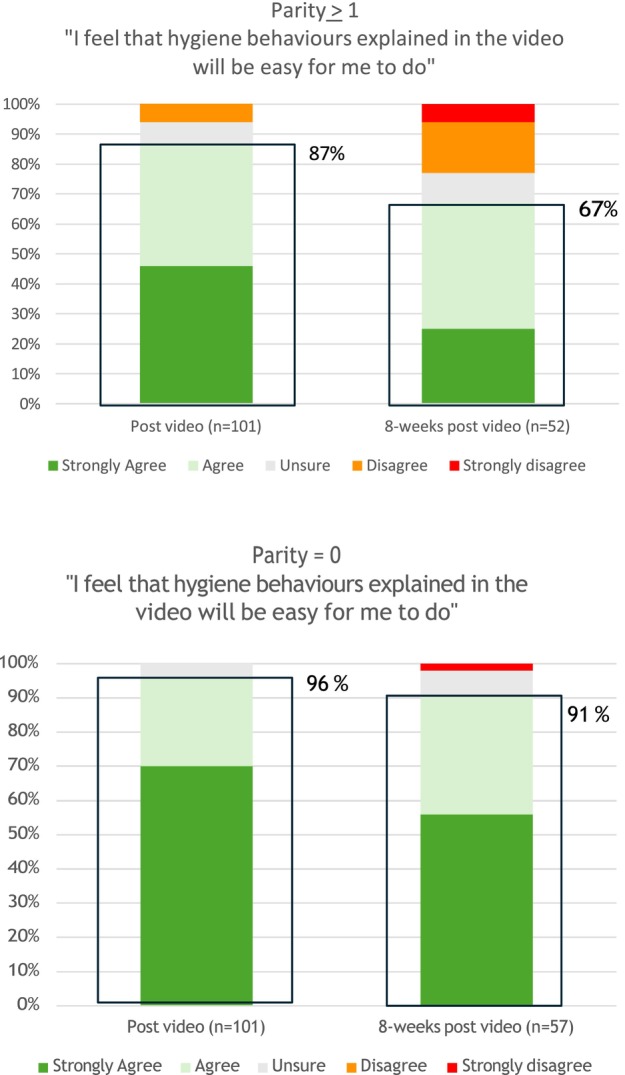
Participants' response to the statement “I feel that hygiene behaviours explained in the video will be easy for me to do” after watching the video and 8 weeks later, categorised by parity.

At T2, 99% of participants agreed the CMV education video provided important information, and 91% felt the hygiene behaviours explained in the video would be easy to follow. However, this proportion decreased to 80% (87/109) at T3, that is, 8 weeks after watching the video. The decline was more pronounced among participants with previous births, dropping from 87% at T2 to 67% at T3 (*p* < 0.01), compared to those without prior births, where the decrease was from 96% to 91% (*p* = 0.19) (Figure 3).

At T2, participants were asked to select from a list to describe how they felt about the information provided in the video (Q8‐Appendix Survey S2). A total of 405 responses were recorded from 202 participants who answered this question. The most common emotions were “informed” (*n* = 165), “worried” (*n* = 89), and “knowledgeable” (*n* = 50).

Free‐text responses received at T2 and T3 were organised into three ICA categories: (1) Considering the importance of the message (e.g., “Urging me to educate family and friends”); (2) Reacting with emotion (e.g., “Sad I wasn't shown this in previous pregnancies. Baby #3 was stillborn due to CMV”); and (3) Perceiving personal barriers to implementation (e.g., “Because toddlers are hard to say no to”) (Table 2).

**TABLE 2:** Qualitative analysis of free text responses.

Statement—“The information provided in the video made me feel”	
Participants response–“Other”	
Time point—Post CMV video (T2)	
Categories	Free text response
Considering the importance of the information	“Made me take CMV risk more seriously” “Urging me to educate my family and friends” “Slight sceptical, seems like a good info, but why have I got this far in life with no knowledge of this if it can have such lasting effects, I'm not sure how real this threat is or whether it is just another thing to be worried about in pregnancy”
Reacting with emotion	“Surprised its not widely spoken about or tested for” “Sad I wasn't shown it in previous pregnancies. baby #3 was stillborn due to CMV” “Anxious” “Concerned I have not undertaken these measures earlier in the pregnancy” “Angry because I can't believe this isn't more widely known and there isn't a vaccine”
Perceiving personal barriers	“How realistic when you have a snotty toddler in daycare haha”

Statement—“CMV hygiene precautions would be easy to implement”	
Participants' response—“Disagree/Strongly disagree”	
Time point—Post CMV video (T2)	
Categories	Free text response
Perceiving personal barrier	“Having a young child you are forever, wiping drool, wiping noses, testing food, cleaning up toys so being able to completely isolate yourself from these behaviours is quite” “Having a toddler who is seeking of affection I can imagine it would be hard to refuse a kiss on the lips and make them kiss my head instead. I also feel sharing food with her is so engrained in our routine that it would be difficult to break.” “Because toddlers are hard to say no to” “1. changing nappies does not always occur near soap and water. this often needs to happen in the boot of a car with nothing but hand sanitizer. 2. in principle not sharing food with a toddler seems fine. in practice anyone with a toddler knows this is almost impossible in practice. 3. washing hands with soap after wiping a toddler's nose is also not practical for the same reasons as the nappy changes”

Statement—“CMV hygiene precautions will be easy for me to do”	
Participants response—“Disagree/Strongly disagree”	
Time point—8‐weeks, post CMV video (T3)	
Categories	Free text response
Perceiving Personal barrier	“Toddlers don't understand personal space and it's not possible or practical to always be able to wash hands” “Because I love my toddler and like to show affection with big cuddles and kisses” “Toddlers get sick so often, I'm constantly wiping noses and washing my hands every time is just not practical” “Impossible to stop my younger toddler giving me kisses and close cuddles”

Abbreviation: CMV, cytomegalovirus.

## 4 Discussion

This is the first study in Australia to demonstrate that a brief educational video can significantly increase pregnant women's knowledge of congenital CMV, its transmission risks, and their willingness to adopt preventative behaviours, with sustained improvements in hygiene practices over 8 weeks. These findings support the potential of short, accessible video‐based interventions to enhance awareness and knowledge about preventable congenital infections. Pregnant women can reduce the risk of CMV infection by practicing good hygiene when caring for young children [8, 9, 17]. Educating all pregnant women on CMV hygiene precautions is considered best practice according to national and international guidelines [12, 13, 14, 15]. However, this is not routinely implemented in antenatal care [16]. Educational videos represent a valuable tool for informing pregnant women about congenital CMV and promoting prevention behaviours while reaching a broad audience.

Our results align with trials in the UK and USA, which found that educational videos effectively improve knowledge and promote hygiene behaviours among pregnant women [20, 25]. The videos were well‐received, and our results further validate this approach for an Australian population. Additionally, studies have shown that various forms of CMV education—whether counselling, factsheets, or videos—can reduce behaviours that increase the risk of infection [26, 27, 28]. One study found videos to have a higher appeal and greater impact on behaviour change than factsheets [26]. Another demonstrated that participants who modified their hygiene practices found the changes easy to implement because they felt informed and empowered [29]. These findings highlight the willingness of pregnant women to protect their unborn child, challenging resistance from some health professionals to CMV education [30].

Our findings underscore the need to provide CMV prevention information to all pregnant women, regardless of prior pregnancy history. We found no significant difference in baseline CMV knowledge based on obstetric history, though those who had heard of CMV or received prior education had higher awareness levels.

Given the proven effectiveness of educational videos, integrating them into clinical settings, waiting rooms, or digital health platforms offers a practical and efficient method to consistently inform pregnant women about CMV. However, maternity care providers must also be supported to have informed discussions about congenital CMV. Our group has demonstrated that flexible online learning courses effectively improve CMV knowledge among GPs and midwives [31, 32, 33]. In 2024, we launched a RANZCOG‐sponsored eLearning course for obstetricians, ensuring multidisciplinary access to free high‐quality CMV education [34].

The longitudinal design of this study is a major strength, allowing for the evaluation of changes in CMV prevention knowledge and behaviours beyond the immediate post‐video period. By surveying participants 8 weeks after watching the CMV video, the study reflects real‐world motivation and capacity to implement recommended hygiene behaviours during pregnancy. Notably, women with prior births found these measures harder to sustain at T3, possibly due to caregiving challenges.

Free‐text responses revealed emotional reactions, practical barriers, and concerns, with some participants expressing anxiety or frustration and sharing personal experiences that underscored the need for early prevention. Challenges like avoiding kisses, cuddles, or sharing food with toddlers highlight the importance of realistic strategies, such as promoting “kisses on the forehead.” Healthcare providers can offer achievable goals and actionable guidance to support adherence without undue anxiety. Other strengths include social media recruitment, which captured diverse participants, and the publicly accessible educational video co‐developed with individuals with lived CMV experience.

Limitations include potential selection bias, as participation was limited to English‐speaking individuals, likely over representing women with higher health literacy and education. However, this aligns with the demographic at the highest risk for primary CMV infection during pregnancy, suggesting that recruitment effectively targeted the appropriate population [35]. The reliance on self‐reported data may have introduced bias, particularly in participants' estimates of their adherence to CMV prevention behaviours. Participant attrition at T2 and T3 is another limitation, as it may have introduced bias; however, a mixed‐effects model with a random intercept was used to account for differences between those who completed follow‐ups and those who did not. Finally, the absence of serial CMV testing in participants limits the ability to measure the direct impact of the educational intervention on infection rates.

Longitudinal studies in Australia are needed to track the serological status of pregnant women who receive CMV education to evaluate the public health impact of video‐based interventions. In line with the Australian Pregnancy Care [12] and RANZCOG [13] guidelines recommending that all pregnant women be advised about CMV prevention, future research could explore integrating video education into routine antenatal care to ensure universal access to CMV prevention information. Additionally, qualitative studies are necessary to understand consumer perspectives on barriers to implementing hygiene behaviours while caring for young children. Understanding the viewpoints of pregnant women who disagreed with the CMV risk prevention recommendations may also help refine the messaging and improve the content of CMV education programs.

## Conflicts of Interest

The authors declare no conflicts of interest.

## Supporting information


Data S1.
