# Supplementary material for: A 2‐Min Cytomegalovirus (CMV) Awareness Video Improves Pregnant Women's Knowledge and Planned Adherence to Hygiene Precautions
Source: Aust N Z J Obstet Gynaecol. 2025 Mar 25;65(5):662–70. doi: 10.1111/ajo.70016 (PMC12723089; doi:10.1111/ajo.70016)

Supplementary Table 1: Participant cytomegalovirus (CMV) knowledge by sociodemographic, previous CMV knowledge and pregnancy history at baseline (T1)

|  |  | **Mean baseline score^1^(SD)** | | ***p-value*** |
| --- | --- | --- | --- | --- |
| **Sociodemographic** | | | | |
| SEIFA Quintile |  | |  |  |
|  | 1-3 (n=89) | | 3.20 (2.56) | 0.224 |
|  | 4-5 (n = 180) | | 3.54 (2.56) |  |
| Age |  | |  |  |
|  | <25 years | | 2.71 (2.56) | 0.188 |
|  | 25-34 years | | 3.23 (2.59) |  |
|  | 35 + years | | 3.78 (2.49) |  |
| **Pregnancy history** | | | | |
| Pregnant previously |  |  | |  |
|  | Yes (n = 168) | 3.58 (2.53) | | 0.160 |
|  | No (n = 102) | 3.14 (2.60) | |  |
| Given birth previously |  | | |  |
|  | Yes (n = 145) | 3.52 (2.53) | | 0.493 |
|  | No (n = 23) | 3.30 (2.61) | |  |
| **Prior CMV knowledge** | | | | |
| Previously heard of CMV |  |  | |  |
|  | Yes (n=177) | 4.55 (1.94) | | **<0.001** |
|  | No (n=83) | 1.26 (2.18) | |  |
| Received CMV education this pregnancy |  |  | |  |
|  | Yes (n = 53) | 5.17 (1.12) | | **<0.001** |
|  | No (n=124) | 4.29 (2.16) | |  |
| Regular contact with children under 5 years of age |  |  | |  |
|  | Yes (n=159) | 3.64 (2.48) | | 0.087 |
|  | No (n=111) | 3.10 (2.65) | |  |

Abbreviation: SEIFA – Socio-Economic Indexed for Areas; CMV - Cytomegalovirus

^1^ The maximum total score for the CMV knowledge questions is 6.

Supplementary Table 2: Behaviours affecting cytomegalovirus (CMV) risk by sociodemographic, prior CMV Awareness, and pregnancy history at baseline (T1)

|  |  | Median baseline score^1^ (minimum, maximum) | *p-value* |
| --- | --- | --- | --- |
| **Sociodemographic** | | | |
| SEIFA Quintile |  |  |  |
|  | 1-3 (n=60) | 20 (12.25) | 0.652 |
|  | 4-5 (n = 99) | 19(10,25) |  |
| Age |  |  |  |
|  | <25 years | 17 (16,25) | 0.188 |
|  | 25-34 years | 19 (10,25) |  |
|  | 35 + years | 20 (10,25) |  |
| **Pregnancy History** | | | |
| Pregnant previously |  |  |  |
|  | Yes (n = 139) | 19 (10,25) | **<0.001** |
|  | No (n = 20) | 23 (15,25) |  |
| Given birth previously |  |  |  |
|  | Yes (n = 132) | 19 (10,25) | **<0.001** |
|  | No (n = 07) | 23 (15,25) |  |
| **Prior CMV knowledge** | | | |
| Previously heard of CMV |  |  |  |
|  | Yes (n=112) | 20 (10,25) | 0.197 |
|  | No (n=47) | 19(10,24) |  |
| Received CMV education this pregnancy |  |  |  |
|  | Yes (n=112) | 21 (14,25) | 0.05 |
|  | No (n=47) | 19 (10,25) |  |

Abbreviation: SEIFA – Socio-Economic Indexed for Areas; CMV - Cytomegalovirus

^1^ The maximum total score for the behaviours affecting CMV questions is 25

Appendix Survey 1 – Baseline questionnaire and Pre-CMV video survey (T1)


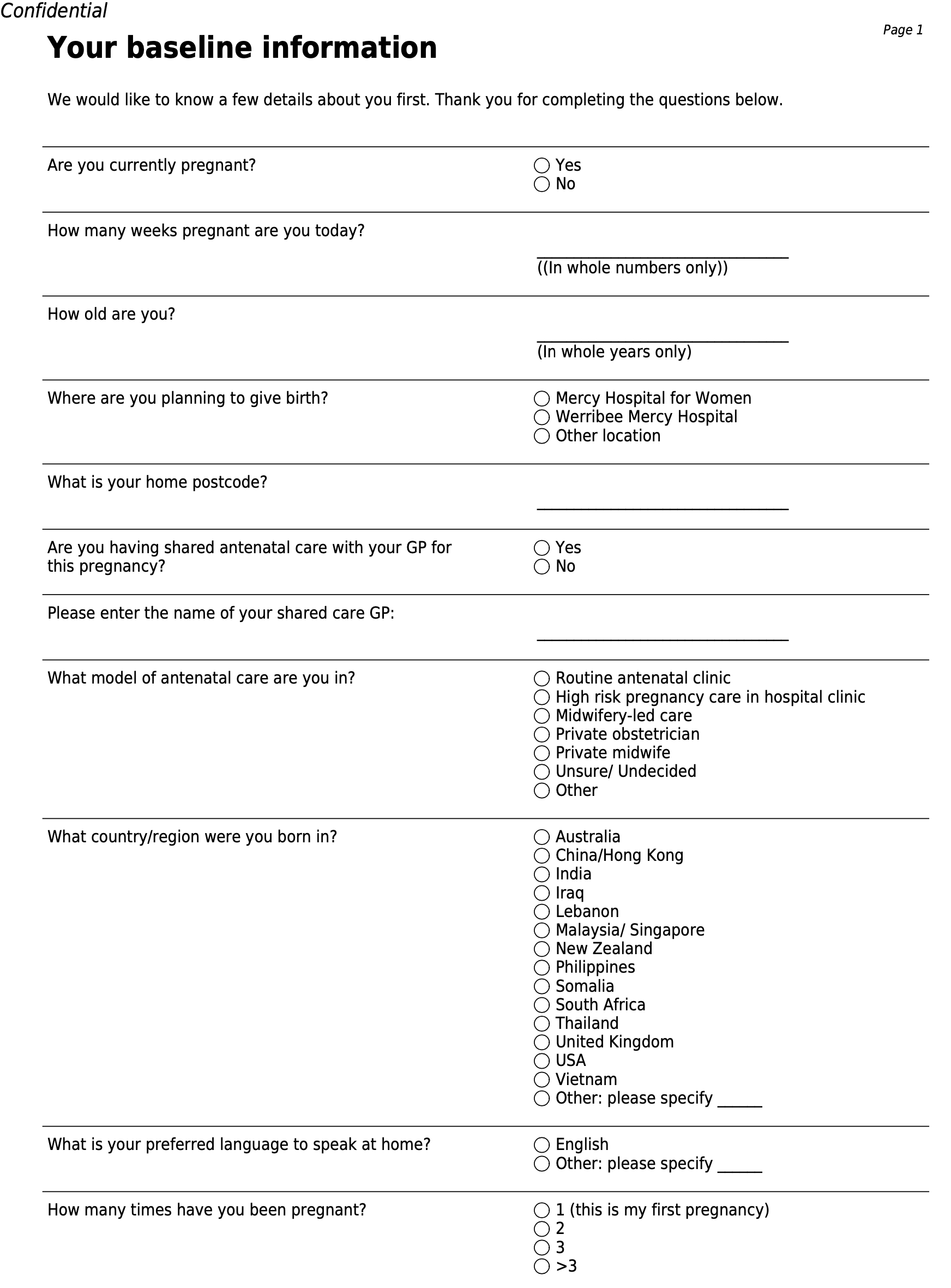


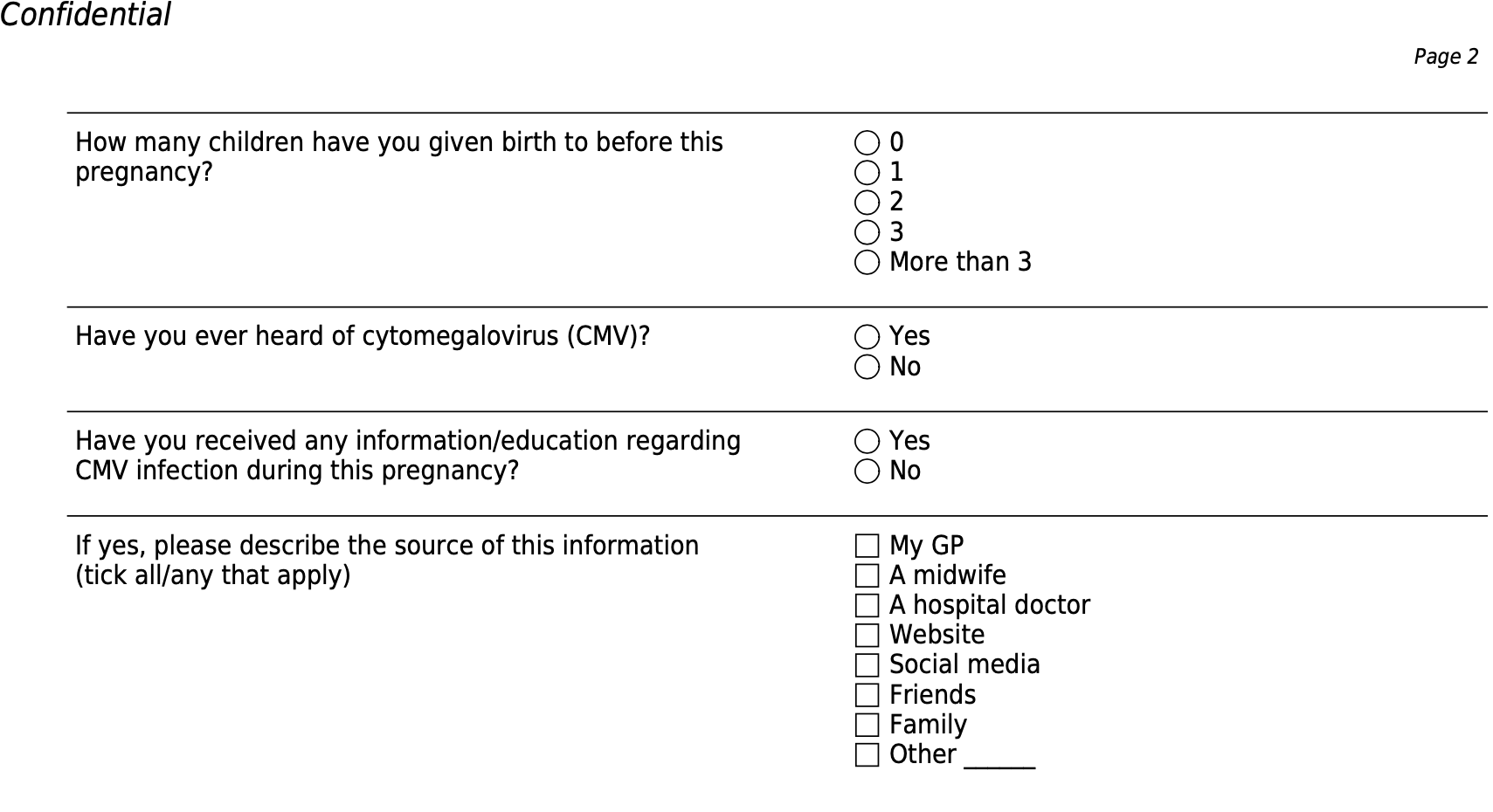


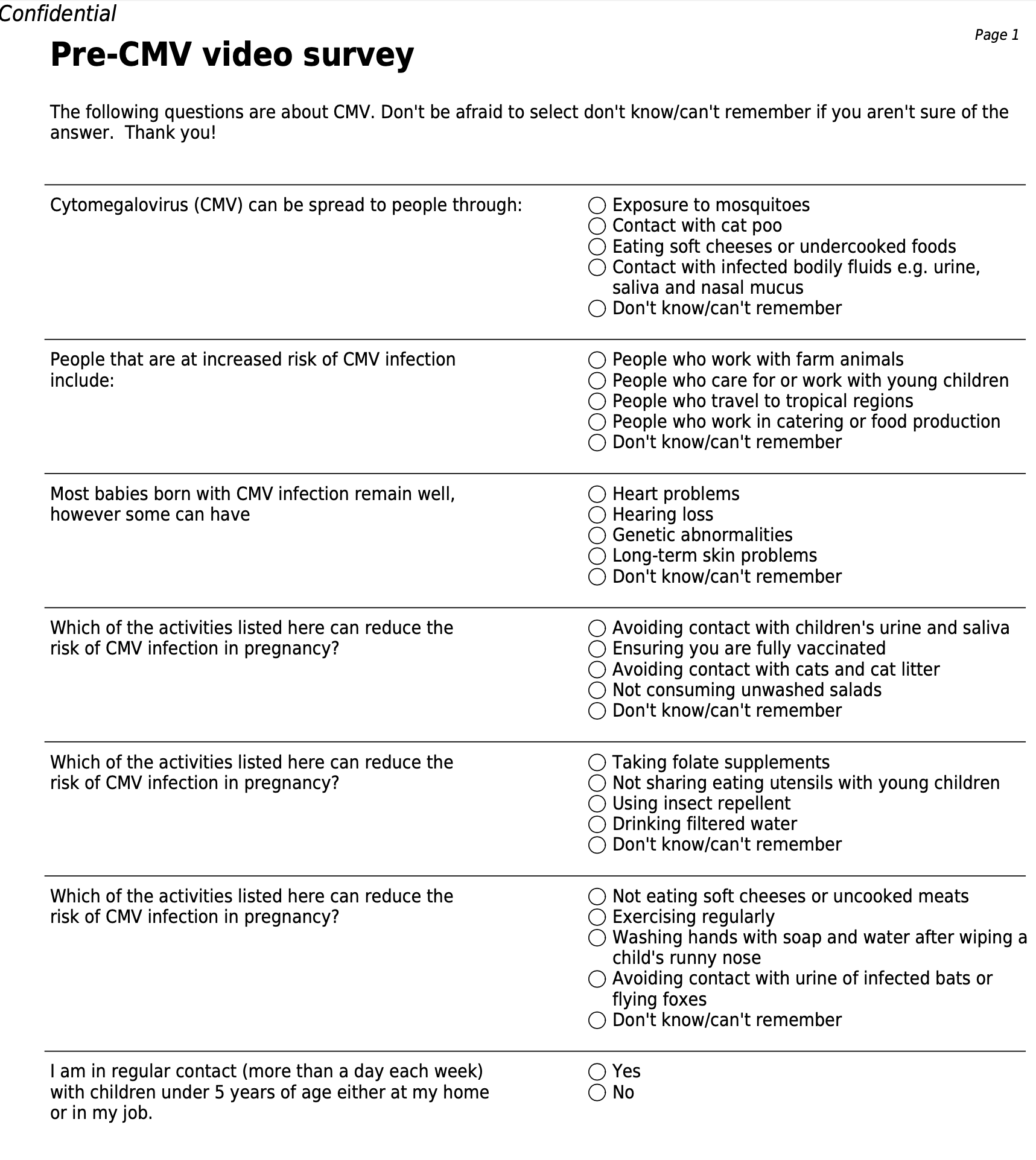


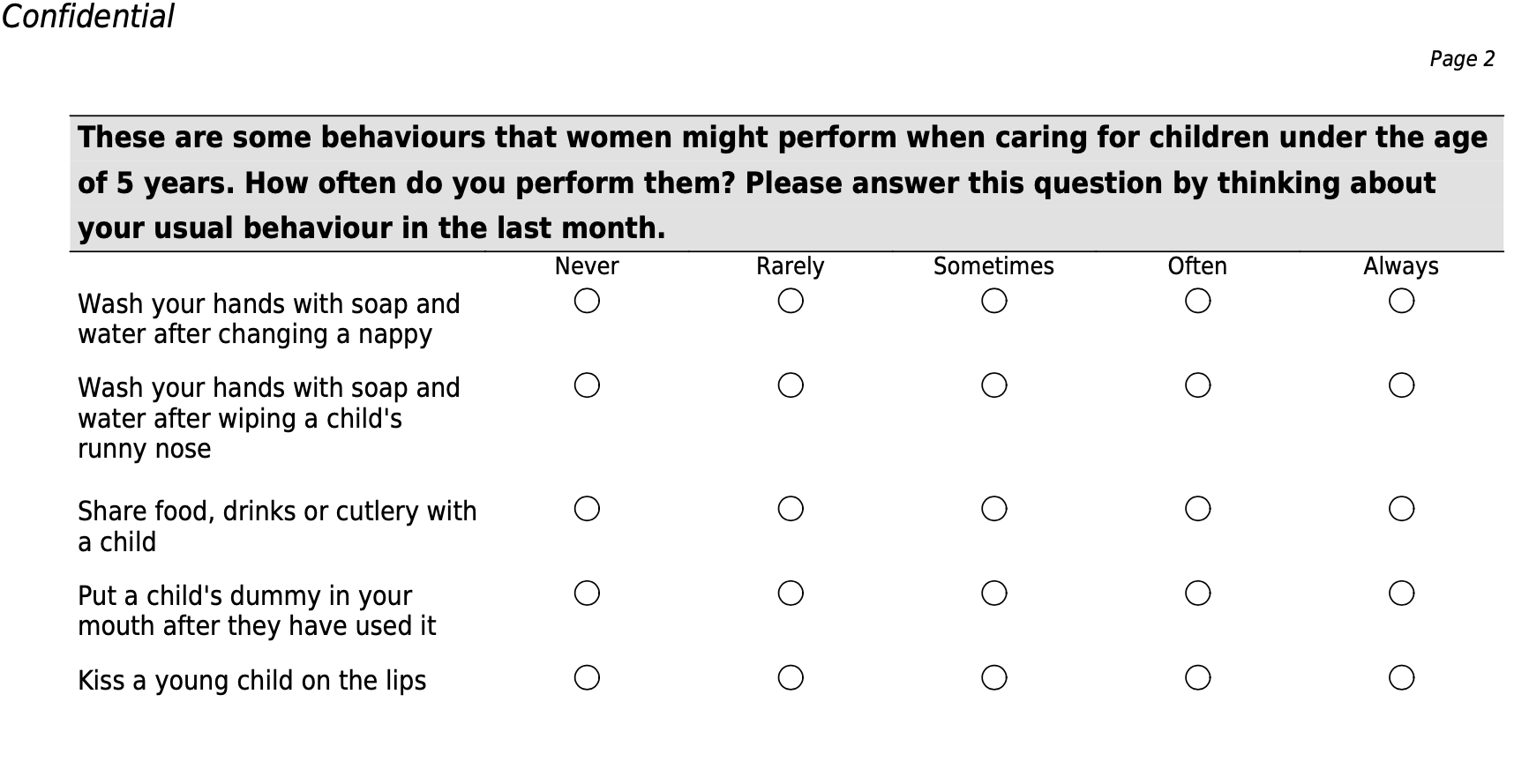


Appendix Survey 2 – Post-video survey (T2)


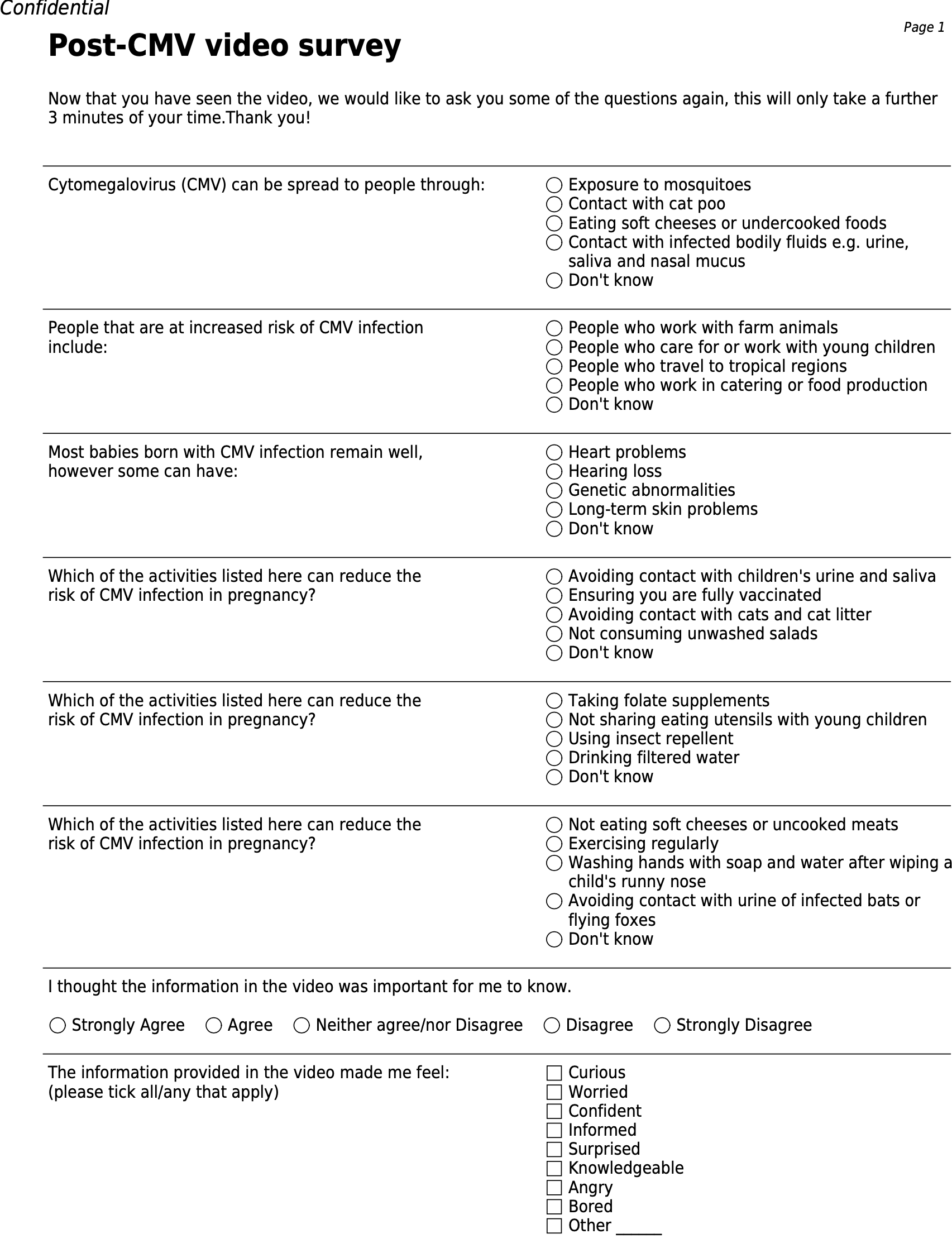


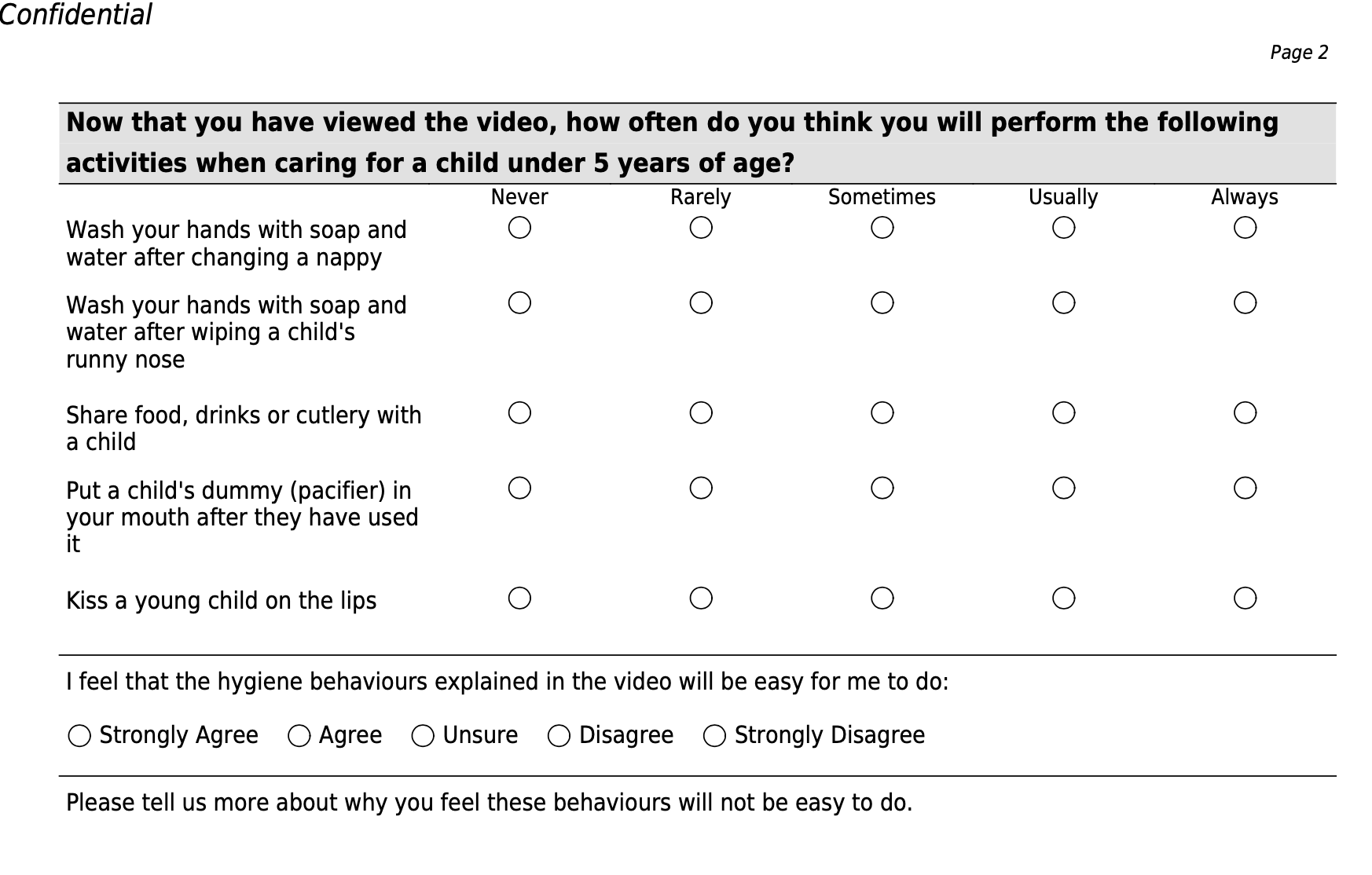


Appendix Survey 3 – Post video, 8-week survey (T3)


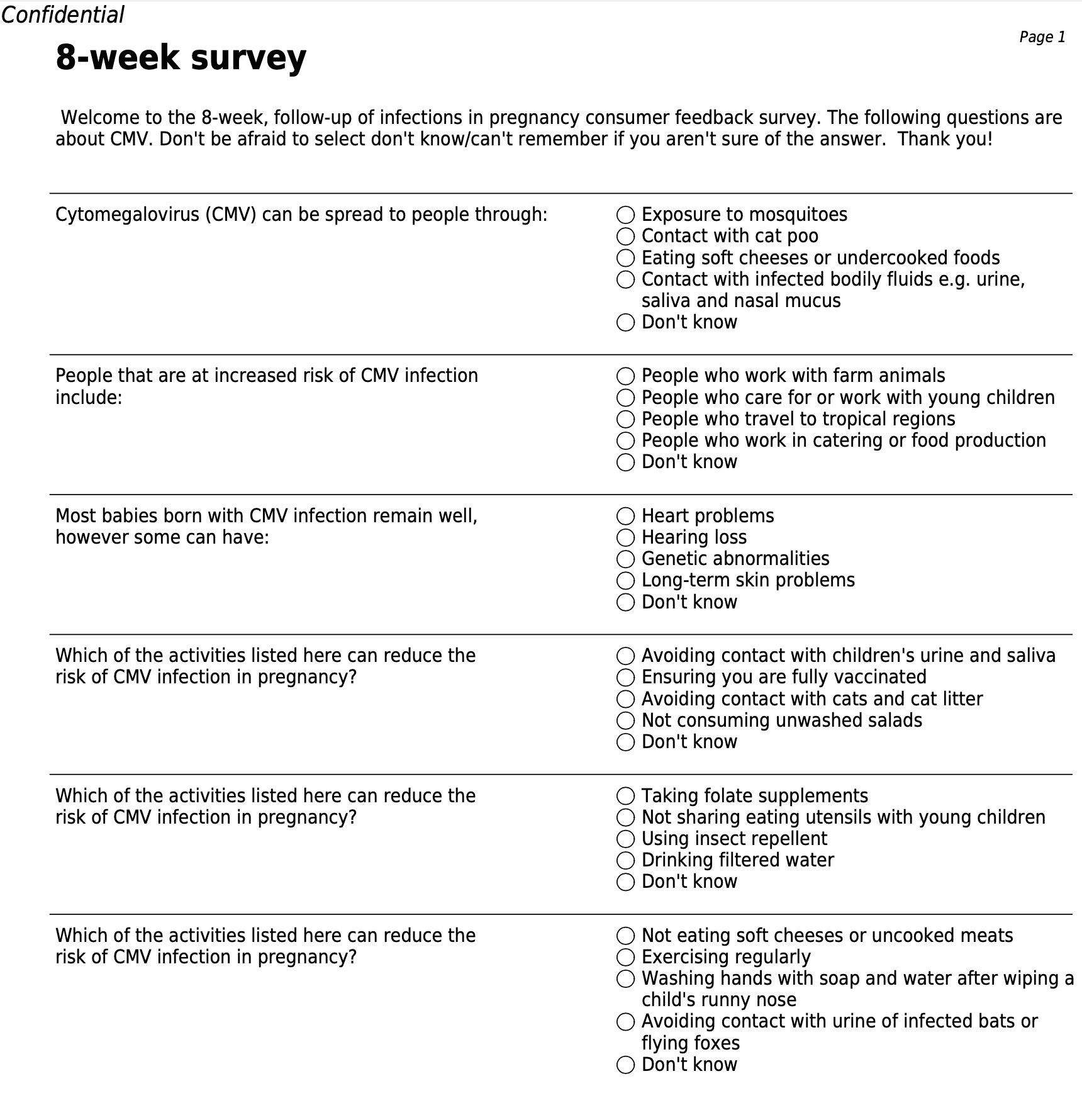


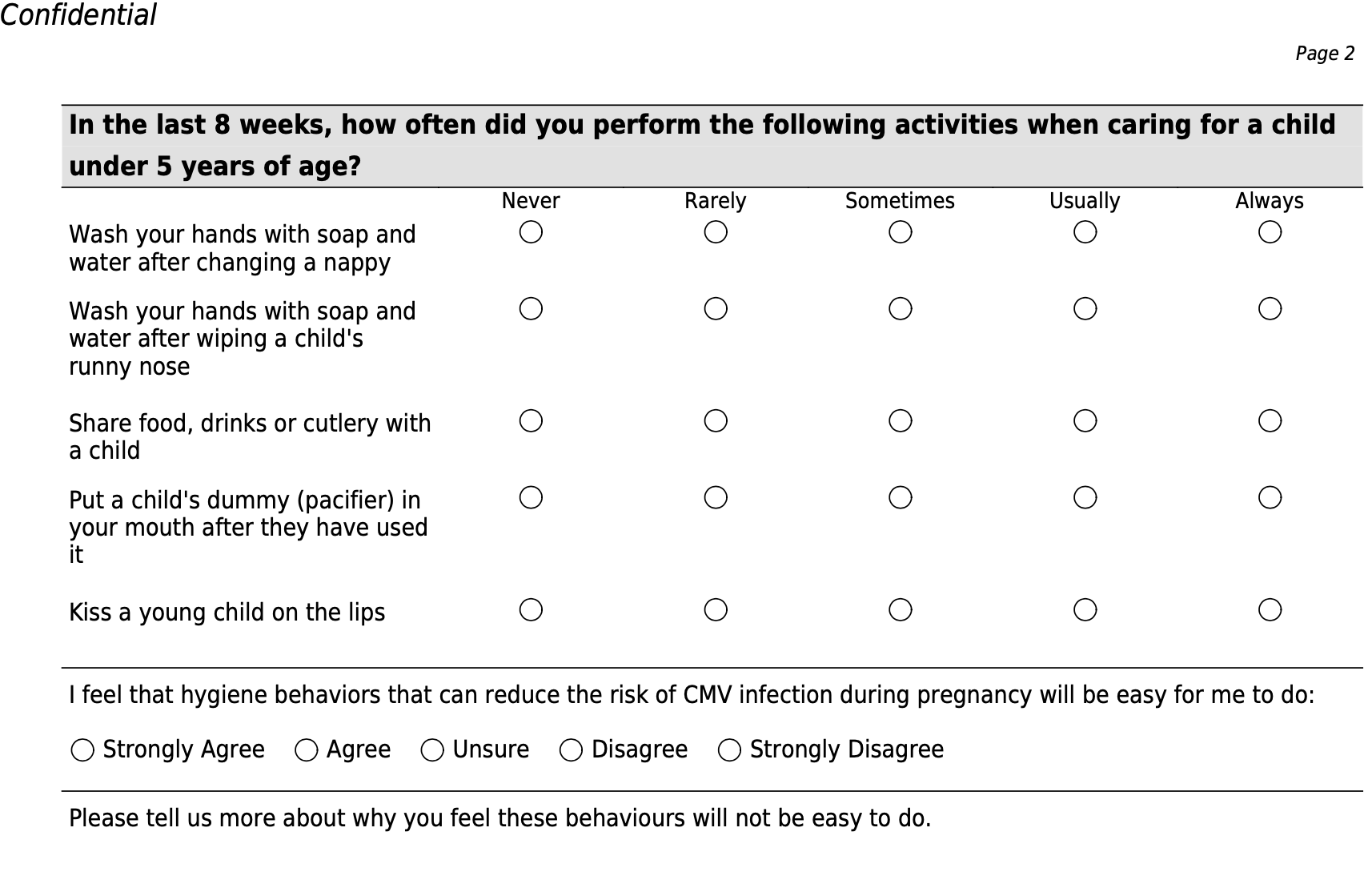

Supplement: Supplementary file 1 — Data S1. [file AJO-65-662-s001.docx]
